# Senkyunolide I Protects against Sepsis-Associated Encephalopathy by Attenuating Sleep Deprivation in a Murine Model of Cecal Ligation and Puncture

**DOI:** 10.1155/2021/6647258

**Published:** 2021-02-15

**Authors:** Jian Xie, Zhen-zhen Zhao, Peng Li, Cheng-long Zhu, Yu Guo, Jun Wang, Xiao-ming Deng, Jia-feng Wang

**Affiliations:** Faculty of Anesthesiology, Changhai Hospital, Naval Medical University, Shanghai, China

## Abstract

Sepsis may lead to sleep deprivation, which will promote the development of neuroinflammation and mediate the progression of sepsis-associated encephalopathy (SAE). Senkyunolide I, an active component derived from an herb medicine, has been shown to provide a sedative effect to improve sleep. However, its role in sepsis is unclear. The present study was performed to investigate whether Senkyunolide I protected against SAE in a murine model of cecal ligation and puncture (CLP). Here, we showed that Senkyunolide I treatment improved the 7-day survival rate and reduced the excessive release of cytokines including TNF-*α*, IL-6, and IL-1*β*. A fear conditioning test was performed, and the results showed that Senkyunolide I attenuated CLP-induced cognitive dysfunction. Senkyunolide I treatment also decreased the phosphorylation levels of inflammatory signaling proteins, including p-ERK, p-JNK, p-P38, and p-P65, and the level of inflammatory cytokines, including TNF-*α*, IL-6, and IL-1*β*, in the hippocampus homogenate. Sleep deprivation was attenuated by Senkyunolide I administration, as demonstrated by the modification of the BDNF and c-FOS expression. When sleep deprivation was induced manually, the protective effect of Senkyunolide I against inflammatory responses and cognitive dysfunction was reversed. Our data demonstrated that Senkyunolide I could protect against sepsis-associated encephalopathy in a murine model of sepsis via relieving sleep deprivation.

## 1. Introduction

Sepsis, termed as life-threatening organ dysfunction induced by polymicrobial infection, is a leading cause of death in the intensive care unit (ICU) [1, 2]. Sepsis may lead to diffuse brain dysfunction in the absence of central nervous system infection, which is also called sepsis-associated encephalopathy (SAE) with a prevalence of up to 70% among patients with severe systemic infection [3, 4]. The clinical manifestations of SAE vary greatly, from compromise in attention and orientation to delirium and even coma [5]. Neuronal apoptosis, microcirculatory dysfunction, and mitochondrial dysfunction have been implicated in SAE [6–8]. The local production of proinflammatory mediators may result in impairment of the central nervous system, and thus, microglia activation and the release of inflammatory cytokines, such as TNF-*α* and IL-1*β*, play a central role in the development of SAE [9, 10].

It has been observed that sleep quality may be impaired in septic rats [11]. In human patients, sepsis might also induce changes in electroencephalogram (EEG) rhythm during sepsis [12]. Therefore, sleep deprivation may be more frequent than we expected during sepsis. It has been reported that sleep deprivation can lead to the release of inflammatory factors, cachexia, and even death in sepsis [13–15]. Therefore, improving sleep quality may be helpful to reduce the level of inflammation and improve the prognosis of sepsis.

Senkyunolide I, which is one of the effective components in an herb medicine named Ligusticum Chuanxiong hort, is mainly distributed in Sichuan province (China) and first recorded in the Divine Husbandman's Classic of the Material Medical (Shen Nong Ben Cao Jing). It has been widely studied because of its antioxidant, analgesic, and sleep-improving effects [16–18]. Tang et al. [19] and Qi et al. [20] reported that SEI could protect cultured PC12 cells and human liver HepG2 cells from oxidative damage induced by hydrogen peroxide. Hu et al. [21] reported that SEI could exert a protective role against focal cerebral I/R injury via its antioxidative and antiapoptotic mechanisms. Additionally, Peng et al. [17] reported that Senkyunolide I provided a sedative effect and was synergetic with pentobarbital in prolonging the sleeping time in mice. In another study performed by Guo and Duan [22], Senkyunolide I was observed to be able to decrease sleep latency in mice. Therefore, we speculated that Senkyunolide I might improve sleep quality and thereby protect against sepsis-induced inflammatory response and brain dysfunction induced by sleep disorders. The present study was performed to investigate the role of Senkyunolide I in the inflammatory level, sleep deprivation, and brain impairment in a murine model of cecal ligation and puncture (CLP).

## 2. Materials and Methods

### 2.1. Animals

C57BL/6J mice (male, 8-10 weeks old) were obtained from GemPharmatech Experimental Animal Corporation (Nanjing, China). All animals were maintained in a room with proper conditions (21°C-23°C, 40%-60% humidity) and a 12 h light/dark cycle. Mice had access to food and water freely. The protocol of the animal study was approved by the ethics committee for Animal Research of Changhai Hospital and conformed to the relevant rules and regulations.

### 2.2. CLP Model

The CLP procedure was performed as previously reported. Generally, mice were anesthetized with 2-3% sevoflurane, and subcutaneous butorphanol (1 mg/kg) was used for analgesia. After skin disinfection, a middle abdominal incision was made and the cecum was exposed. In CLP mice, the cecum was ligated at half of the root and punctured through with a 22G needle. Some cecal content was squeezed out, and the cecum was returned to the abdominal cavity. In the sham-operated mice, the cecum was exposed without any treatment. Then, the skin was closed in one layer, and 1 ml saline was injected subcutaneously for fluid resuscitation.

For most of the studies, mice were euthanized at 24 h after operation for sample harvesting, including the hippocampus, cerebral cortex, and the whole blood obtained by heart puncture. For survival analysis, mortality was observed for seven consecutive days.

### 2.3. Fear Conditioning (FC) Test

A fear conditioning device (XR-XZ301, Xinruan Information Technology, Shanghai, China) was used to test the conditional memory as reported by Ji et al. [23]. Briefly, at the training stage, mice were exposed in the chamber for 3 min, followed by stimulation with a tone (30 s, 65 dB, 3 kHz) and a foot electrical stimulus (3 s, 0.75 mA). The contextual and tone fear conditioning test was carried out 24 h later. Freezing time to context and tone during the 3-minute period was recorded, respectively. The freezing behavior is defined as no visible movement other than respiration.

### 2.4. Sleep Deprivation (SD) Model

A new sleep deprivation device consisting of a cylinder and an inbuilt interfering rod was used for mice (XR-XS108, Xinruan Information Technology, Shanghai, China). The mice were allowed to roam freely in their cylinders, without limitation to food and water. In order to avoid sleeping, the interfering rod rotates consistently at a constant speed (15 rpm) in the chamber to accomplish the sleep deprivation process. The process of sleep deprivation began from the time of full recovery from anesthesia and lasted for 24 h.

### 2.5. Drug Treatment and Groups

**Figure 1:**
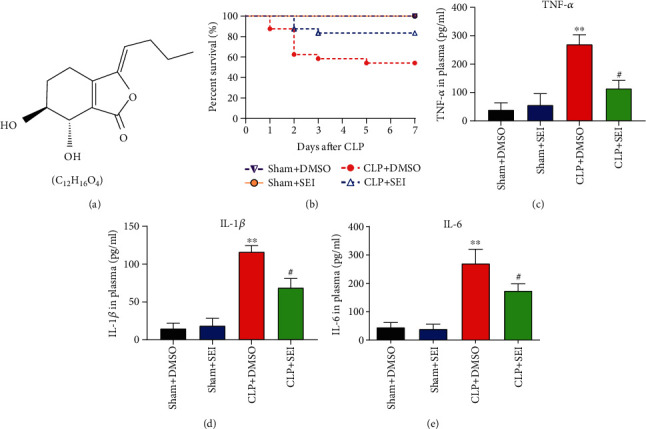
Senkyunolide I treatment improved the survival rate and reduced systemic inflammation of septic mice. (a) The chemical structure and formula of Senkyunolide I. (b) Survival analysis of CLP mice (*n* = 24 for each group, *P* = 0.0048 for CLP+SEI vs. CLP+DMSO). (c–e) Plasma levels of TNF-*α* (c), IL-1*β* (d), and IL-6 (e). Data were displayed as the mean ± SEM (*n* = 6 for each group). ^∗∗^*P* < 0.01 vs. sham groups, #*P* < 0.05 vs. CLP+DMSO group. SEI: Senkyunolide I; CLP: cecal ligation and puncture.

Senkyunolide I (C12H16O4, CAS, 94596-28-8, purity ≥ 98%) was purchased from Zheyan Biotechnology (Shanghai, China). The formula is shown in Figure 1(a). Senkyunolide I was dissolved in dimethyl sulfoxide (DMSO, at a concentration of less than 5%) for animal experiments and injected intraperitoneally (36 mg/kg). The dose was determined in accordance with previous studies [21].

For the pharmacological experiments, the mice were allocated equally into four groups, including the sham+DMSO group, the sham+SEI group, the CLP+DMSO group, and the CLP+SEI group. The mice were administered with DMSO solution or Senkyunolide I solution dissolved in DMSO according to the grouping. For survival analysis, 96 mice were used with 24 mice in each group.

### 2.6. Immunofluorescence Assay

The hippocampal tissues were harvested and cut into paraffin sections which were used for the immunofluorescence assay. Paraffin-embedded hippocampal tissue was cut into a thickness of 10 *μ*m on glass slides, which were then blocked with 10% donkey serum for 60 minutes and incubated with primary antibody (mouse anti-IBA-1, Proteintech, Illinois, USA) at 4°C overnight. The secondary antibody was incubated for 1.5 h in room conditions. The nuclear staining was labeled with 4′,6-diamidino-2-phenylindole (DAPI). Finally, the average IBA-1 number of positive staining in three fields under a fluorescence microscope (Leica, Wetzlar, Germany) and five sections of each group was analyzed.

### 2.7. TUNEL Staining

To investigate whether Senkyunolide I treatment could reduce the apoptotic rate of the neurons, apoptosis in the hippocampus was detected by the TUNEL assay (Roche, Basel, Switzerland). Briefly, the hippocampus tissues were harvested 24 hrs after operation and fixed in 4% paraformaldehyde for each section. When the sections were prepared, they were deparaffinized, hydrated, and treated with proteinase K. Then, the sections were covered with 3% H_2_O_2_ for 5 min at room temperature to block endogenous peroxidase and incubated with terminal deoxynucleotidyl transferase reaction mixture while TBS solution was alternatively applied in the sham group. Finally, we incubated the sections with streptavidin-HRP and diaminobenzidine and counterstained with hematine. The ratio of TUNEL-positive cells in 5 fields in each section was used for the following statistical analysis.

### 2.8. Enzyme-Linked Immunosorbent Assay (ELISA)

The concentrations of inflammatory mediators in samples of serum and hippocampus were quantified using ELISA kits (Thermo, Massachusetts, USA). The hippocampus was homogenized with cold normal saline and centrifuged at 10,000 × g for 10 min to obtain supernatants. Similarly, whole blood was centrifuged at 1500 rpm for 10 min, and the serum was collected. The procedures of the ELISA assay were performed with the instructions provided by the manufacturer (Invitrogen, Carlsbad, CA, USA). The results were read at 450 nm with a spectrophotometer (Synergy2, BioTek, USA).

### 2.9. Western Blot Assay

Proteins in hippocampal tissues were extracted with RIPA lysis buffer encompassing a protease inhibitor cocktail, and the protein concentrations were measured with the BCA assay (Thermo, Massachusetts, USA). The denatured protein samples (30 *μ*g per sample) were separated by 10% SDS-PAGE gel and transferred to polyvinylidene difluoride (PVDF) membranes (Merck, Darmstadt, Germany). The transferred membranes were blocked with 5% skimmed milk and then incubated with primary antibody at 4°C overnight. The HRP-conjugated secondary antibody was added to incubate the washed membrane for 3 h at room temperature. Finally, the membranes were visualized with the ECL reagent (Thermo, Massachusetts, USA). The protein bands were analyzed by using ImageJ software (National Institutes of Health, Maryland, USA). The primary antibodies included p-ERK (1 : 1000, CST, Boston, USA), ERK (1 : 1000, CST, Boston, USA), p-JNK (1 : 1000, CST, Boston, USA), JNK (1 : 1000, CST, Boston, USA), p-P38 (1 : 1000, CST, Boston, USA), P38 (1 : 1000, CST, Boston, USA), p-P65 (1 : 1000, CST, Boston, USA), P65 (1 : 1000, CST, Boston, USA), BDNF (1 : 1000, Abcam, Massachusetts, USA), c-FOS (1 : 1000, CST, Boston, USA), and beta-actin (1 : 2000, Sigma, USA).

### 2.10. Statistical Analysis

All data were statistically analyzed using the GraphPad Prism 8.4 software (La Jolla, CA, USA). The data were presented as mean values ± standard error of mean and compared using one-way ANOVA, with a post hoc analysis of Tukey's multiple comparisons for homogeneity of variance or Tamhane's T2 test for heterogeneity of variance. The survival rates were compared using the log-rank analysis. A *P* value < 0.05 was considered statistically significant.

## 3. Results

### 3.1. Senkyunolide I Improved Survival Rates and Reduced Systemic Inflammation in Septic Mice

To investigate the effects of Senkyunolide I on the overall survival of mice, the CLP mice were administered with Senkyunolide I (36 mg/kg) and observed for 7 consecutive days. In the CLP+DMSO group, 13 of 24 mice survived to the seventh day (Figure 1(b)), while Senkyunolide I administration could significantly improve the survival rates of CLP mice (-20 of 24 mice survived, Figure 1(a), *P* = 0.0048).

ELISA was performed to analyze proinflammatory factors including TNF-*α*, IL-1*β*, and IL-6 in serum. The serum levels of TNF-*α* (Figure 1(c)), IL-1*β* (Figure 1(d)), and IL-6 (Figure 1(e)) were significantly increased at 24 h after CLP operation. Compared with the CLP+DMSO group, Senkyunolide I treatments remarkably decreased the concentrations of TNF-*α*, IL-1*β*, and IL-6 in plasma. These results indicated that Senkyunolide I administration after CLP operation was protective against sepsis challenge.

### 3.2. Senkyunolide I Attenuated the Memory Impairment in CLP Mice

**Figure 2:**
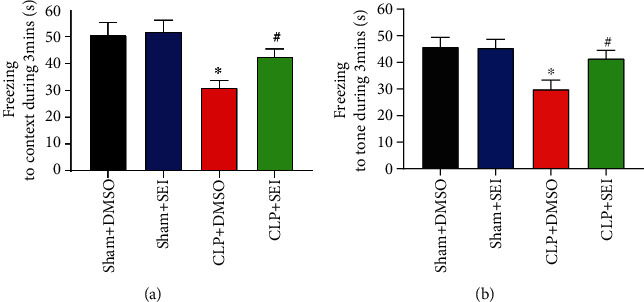
Senkyunolide I treatment protected against cognitive dysfunction in septic mice. (a) Freezing to context and (b) freezing to tone were tested at 24 h after surgery, respectively. Data were shown as the mean ± SEM (*n* = 8 for each group). ^∗^*P* < 0.05 vs. sham groups. #*P* < 0.05 vs. CLP+DMSO group. SEI: Senkyunolide I; CLP: cecal ligation and puncture.

The fear conditioning test was performed to assess the effects of Senkyunolide I on CLP-induced memory impairment. As shown in Figures 2(a) and 2(b), the time of freezing to context and the time of freezing to tone were significantly declined in the CLP+DMSO group than in their counterparts in the sham group at 24 h, whereas Senkyunolide I treatment attenuated the decline in the CLP+DMSO group. Thus, Senkyunolide I may have prevented CLP-induced memory impairment.

### 3.3. Senkyunolide I Alleviated Apoptosis and Suppressed the Activation of Microglia in the Hippocampus Region of Sepsis Mice

**Figure 3:**
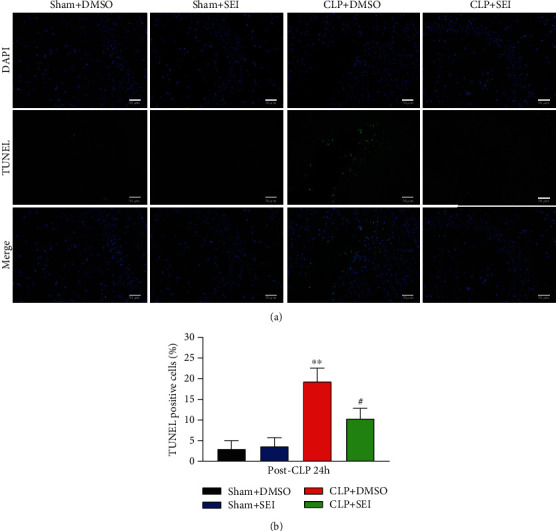
Senkyunolide I administration significantly reduced the number of apoptotic cells in the hippocampus of septic mice. (a) Representative images of TUNEL fluorescence of coronal sections of the hippocampus at 24 h after CLP surgery. (b) Quantification of TUNEL-positive cells. Values were shown as the mean ± SEM (*n* = 6 for each group). ^∗∗^*P* < 0.01 vs. sham groups; #*P* < 0.05 vs. CLP group. SEI: Senkyunolide I; CLP: cecal ligation and puncture. Scale bar = 50 *μ*m.

**Figure 4:**
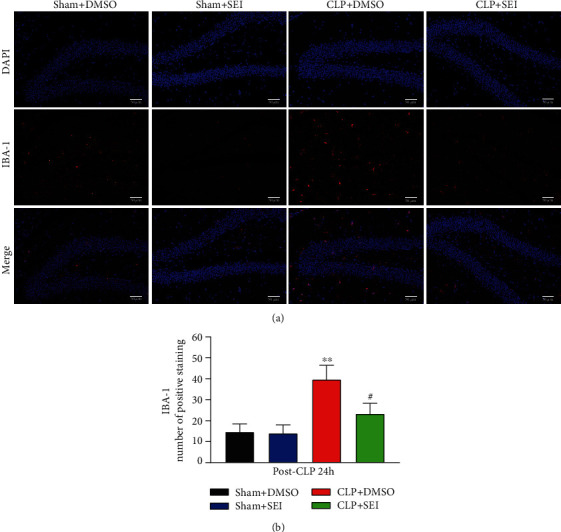
Sepsis-induced IBA-1 activation in the hippocampus was attenuated by Senkyunolide I treatment. (a) Representative images of IBA-1 expression at 24 h after CLP surgery. (b) Bar graphs indicated the number of IBA-1-labeled microglia in the four groups. Data were presented as the mean ± SEM (*n* = 6 for each group). ^∗∗^*P* < 0.01 vs. sham groups, #*P* < 0.05 vs. CLP group. SEI: Senkyunolide I; CLP: cecal ligation and puncture. Scale bar = 50 *μ*m.

The TUNEL assay was performed to examine the neuroprotective effects of Senkyunolide I. It was shown that the rates of apoptotic cells were greatly increased under sepsis challenge, while Senkyunolide I treatment significantly inhibited neuronal apoptosis as shown in Figures 3(a) and 3(b). Microglia activation is a marker for neuroinflammation in SAE, which may be associated with neuronal injury. Therefore, we detected IBA-1, a marker of activated microglia, by the immunofluorescence assay. The fluorescence intensity of IBA-1 was significantly higher in the CLP+DMSO group than in the sham group, while Senkyunolide I treatment surely reversed these changes (Figures 4(a) and 4(b)), suggesting that Senkyunolide I may suppress microglial activation to inhibit neuroinflammation.

### 3.4. Senkyunolide I Inhibited the Activation of the Inflammatory Signaling Pathways in the Hippocampus

**Figure 5:**
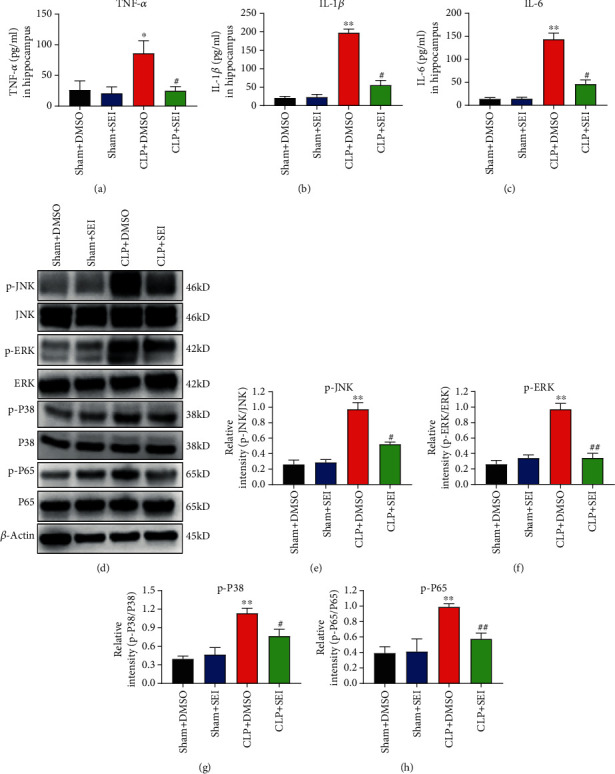
Sepsis-induced neuroinflammation was attenuated by Senkyunolide I treatment. (a) TNF-*α*, (b) IL-1*β*, and (c) IL-6 in the hippocampus homogenate were measured at 24 h after CLP surgery. (d) Representative bands showed the levels of p-JNK, JNK, p-ERK, ERK, p-P38, P38, p-P65, and P65 protein in hippocampus tissue at 24 h after surgery. The relative quantification of p-JNK/JNK (e), p-ERK/ERK (f), p-P38/P38 (g), and p-P65/P65 (h) was analyzed. Values were the mean ± SEM (*n* = 6 for each group). ^∗∗^*P* < 0.01 vs. sham groups; *^##^P* < 0.01 vs. CLP+DMSO group; #*P* < 0.05 vs. CLP+DMSO group. SEI: Senkyunolide I; CLP: cecal ligation and puncture.

The MAPK signaling pathway and NF-*κ*B signaling pathway are involved in various inflammatory diseases and contribute to the production of TNF-*α*, IL-1*β*, and IL-6, hence reducing neuronal plasticity and neurogenesis [24]. These inflammatory signaling pathways were also demonstrated to be activated in the process of sepsis-associated encephalopathy [25, 26]. Therefore, we detected the expression of inflammatory factors and the activation status of these signaling molecules in the hippocampus homogenate. The elevation of TNF-*α* (Figure 5(a)), IL-1*β* (Figure 5(b)), and IL-6 (Figure 5(c)) in CLP mice was attenuated by Senkyunolide I administration. Similarly, the phosphorylation levels of JNK, ERK, p38, and p65 were all enhanced in CLP mice, but Senkyunolide I reduced the activation level significantly as shown in Figures 5(d)–5(h).

### 3.5. The Anti-inflammatory Effect of Senkyunolide I Depended on the Attenuation of Sleep Deprivation during Sepsis

**Figure 6:**
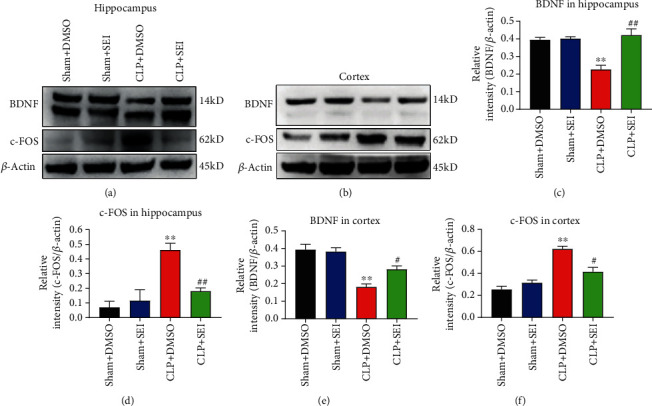
Sleep deprivation of septic mice was attenuated by Senkyunolide I treatment. (a, b) Sleep deprivation markers BDNF and c-FOS in both hippocampus and cerebral cortex were measured by western blot assay. (c–f) The relative quantification of BDNF/*β*-actin and c-FOS/*β*-actin in both hippocampus and cerebral cortex was analyzed. Values were presented as the mean ± SEM (*n* = 6 for each group). ^∗∗^*P* < 0.01 vs. sham groups; *^##^P* < 0.01 vs. CLP+DMSO group; #*P* < 0.05 vs. CLP+DMSO group. SEI: Senkyunolide I; CLP: cecal ligation and puncture.

Some studies have confirmed that sleep deprivation is associated with immune dysfunction, inflammatory cytokine release, cachexia, and other adverse events; thus, treatment targeting sleep deprivation may be helpful in attenuating SAE [15, 27]. Since BDNF and c-FOS are markers related to sleep deprivation [28], we investigated whether Senkyunolide I can modulate the expression of these two proteins. The western blot assay revealed that the expression level of BDNF was highly decreased, while the c-FOS level was significantly elevated in both the hippocampus and cerebral cortex in septic mice. However, Senkyunolide I treatment could reverse these alterations of BDNF and c-FOS induced by CLP (Figures 6(a)–6(f)).

**Figure 7:**
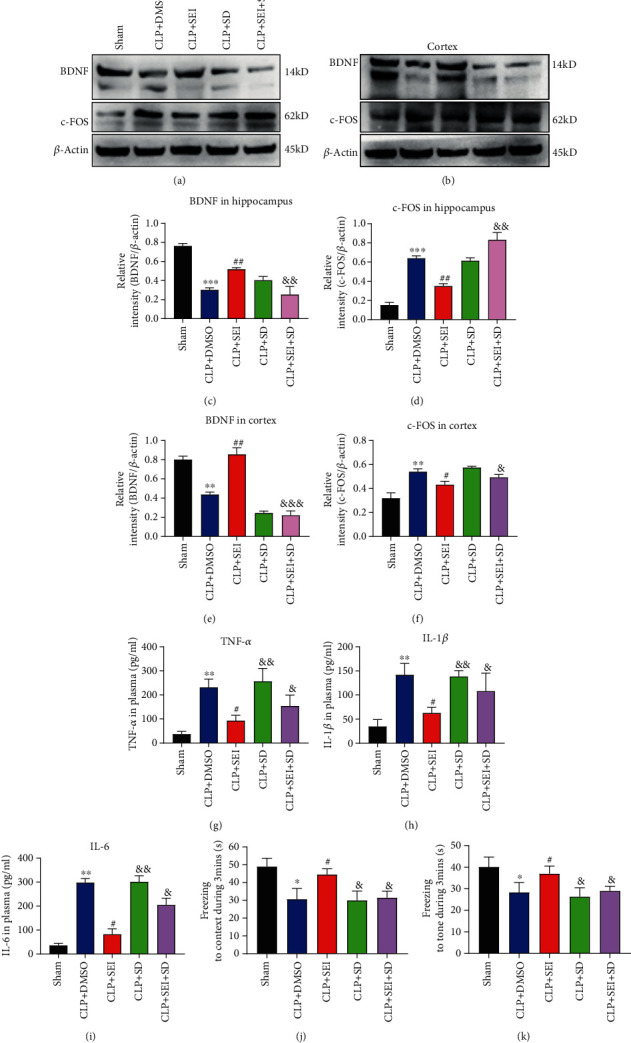
Improvement of cognitive dysfunction by Senkyunolide I of septic mice was reversed by sleep deprivation. Sleep deprivation was induced for 24 h after surgery. (a, b) Expression levels of BDNF and c-FOS were detected by western blot. (c–f) The relative quantification of BDNF/*β*-actin and c-FOS/*β*-actin in both hippocampus and cerebral cortex was analyzed. The levels of TNF-*α* (g), IL-1*β* (h), and IL-6 (i) in the serum were analyzed by ELISA. Freezing to context (j) and freezing to tone (k) were tested at 24 h after surgery, respectively. Values were presented as the mean ± SEM (*n* = 6-8 for each group). ^∗^*P* < 0.05 vs. sham groups, ^∗∗^*P* < 0.01 vs. sham groups, and ^∗∗∗^*P* < 0.001 vs. sham groups; #*P* < 0.05 vs. CLP+DMSO groups, *^##^P* < 0.01 vs. CLP+DMSO groups; *^&^P* < 0.05 vs. CLP+SEI group, *^&&^P* < 0.01 vs. CLP+SEI group. SEI: Senkyunolide I; CLP: cecal ligation and puncture.

A cylinder device with an interfering rod was used to induce sleep deprivation to investigate whether attenuation of sleep deprivation was involved in the protective role of Senkyunolide I on the activation of inflammatory responses. As revealed in Figures 7(a)–7(f), the effect of Senkyunolide I on the expression of BDNF and c-FOS was reversed by induced sleep deprivation. Similar results were also observed in the plasma levels of proinflammation cytokines that sleep deprivation diminished the protective role of Senkyunolide I against proinflammatory responses induced by CLP (Figures 7(g)–7(i)). Furthermore, the fear conditioning test revealed that the freezing time to both of the context and tone returned to the level of CLP mice even if Senkyunolide I was administered (Figures 7(j) and 7(k)).

## 4. Discussion

In the present study, we investigated the therapeutic effect of Senkyunolide I on sepsis and SAE in a murine CLP model. Our data suggested that Senkyunolide I could improve survival and inhibit systemic inflammation in CLP mice. Sepsis-induced memory impairment was attenuated by Senkyunolide I. Senkyunolide I reduced apoptosis, microglia activation, and the activation of MAPK and NF-*κ*B signaling pathway in the hippocampus region. The alteration of the markers of sleep deprivation was attenuated by Senkyunolide I, including the downregulation of BDNF and upregulation of c-FOS. When sleep deprivation was induced, the protective role of Senkyunolide I against systemic inflammation and memory impairment was reversed partially or completely.

Ligusticum Chuanxiong hort belongs to the genus Ligusticum, which includes 66 species of flowering plants. It is popular in treating cerebrovascular diseases in China such as ischemic stroke and migraine [29]. Senkyunolide I, one of the main active ingredients of Ligusticum Chuanxiong hort, has good lipid solubility and can be absorbed quickly into the blood and cerebrospinal fluid. Many Senkyunolide isomers have been proved to be antioxidative agents to inhibit the formation of ROS and lipid peroxidation and act as OH scavenger, among which Senkyunolide I has been demonstrated to show anti-inflammation, antiapoptosis, and antioxidative effects on several studies of neural injury. Hu et al. [21, 30] reported that Senkyunolide I could alleviate stroke-induced neuroinflammation via suppressing the TLR4/NF-*κ*B pathway and protect against cerebral reperfusion injury with its antiapoptosis properties. The antioxidative nuclear factor, Nrf2, was demonstrated to be activated by Senkyunolide I, with the upregulation of heme oxygenase-1 [21]. Another study showed that Senkyunolide I could reduce the sleep latency and exert a synergetic sleep-promoting effect [17]. However, the role of Senkyunolide I on SAE is seldom reported.

Inflammation plays a vital role in the pathogenesis of sepsis. The bacteria themselves and their metabolites can elicit an exorbitant inflammatory response and lead to profound release of inflammatory mediators, such as TNF-*α*, IL-6, and IL-1*β*. The overreleased cytokines positively impact the evolvement of sepsis and even result in organ damage and finally lead to death [31]. Our data demonstrated that the concentrations of proinflammatory cytokines were indeed elevated in the CLP+DMSO group compared with those in the sham groups in both serum and hippocampus homogenates. Senkyunolide I administration significantly reduced the levels of these cytokines and thereby improved the survival rate of septic mice.

Brain dysfunction induced by sepsis, which is also called SAE, is characterized by neuronal apoptosis and microbial activation [32, 33]. Excessive metabolism of oxygen may be present during sepsis, leading to cytochrome c release, caspase-3 activation, and ultimately apoptosis [22]. The number of TUNEL-positive cells was significantly reduced by Senkyunolide I in the hippocampus at 24 h after CLP operation, suggesting that Senkyunolide I attenuated apoptosis in the hippocampus. Furthermore, Senkyunolide I treatment suppressed the expression of IBA-1 and the release of IL-1*β* in the hippocampus, which was a marker of microglial activation. Henry et al. [34] reported that systemic inflammation induced by peripheral inflammatory stimuli could lead to microglial activation and thus facilitate the release of IL-1*β* into the CNS. Another study showed that inhibition of microglial activation could decrease oxidative stress and cytokine levels in the hippocampus, which would improve the cognitive behavior of septic mice [9]. Further assays showed that activation of proinflammatory signaling pathways (NF-*κ*B and MAPK) was also inhibited in the hippocampus. These data suggested that Senkyunolide I had a neuroprotective effect against SAE.

The potential mechanism of the neuroprotective effect of Senkyunolide I remains unclear. It was interesting to notice that BDNF was reduced while c-FOS was elevated in septic mice, which indicated that sleep deprivation was present in septic mice [28]. Sleep deprivation might activate microglia and promote the release of inflammatory mediators [35]. Other studies also reported that sleep deprivation would result in immune dysfunction and impair the capacity of host defense [13, 15, 27]. Therefore, it was not strange that Senkyunolide I attenuated sepsis-induced memory impairment when it reversed the alterations of the markers of sleep deprivation. The role of sleep deprivation seemed to be critical for Senkyunolide I, since induced sleep deprivation could reverse the protective effect of Senkyunolide I.

## 5. Conclusion

In conclusion, Senkyunolide I can alleviate sepsis-induced mortality and systemic inflammation. Senkyunolide I may also be protective against sepsis-associated encephalopathy as demonstrated by apoptosis, microglial activation, and activation of MAPK and NF-*κ*B signaling pathways. Sleep deprivation is attenuated by Senkyunolide I and may participate in an important role of Senkyunolide I in protecting against sepsis-associated encephalopathy.

## Data Availability

The datasets during and/or analyzed during the current study are available from the corresponding authors on reasonable request.
